# Microstructure and Dynamic Properties of CrMnFeCoNi(Al)_8_ Laser Cladding Coatings on Urban Rail Wheels

**DOI:** 10.3390/ma19061173

**Published:** 2026-03-17

**Authors:** Xu Zhang, Peixin Wei, Yuqing Wang, Bingzhi Chen, Wenfang Dong, Xianglong Cao

**Affiliations:** 1 School of CRRC, Dalian Jiaotong University, Dalian 116028, China; 86578692@163.com (X.Z.); chenbingzhi06@hotmail.com (B.C.); m17686490531@163.com (W.D.); 2 Nanjing Conney Electromechanical Co., Ltd., Nanjing 210039, China; yuqing202602@163.com (Y.W.); caoxl2026@163.com (X.C.)

**Keywords:** CrMnFeCoNi(Al)_x_, high-entropy alloy, laser cladding, CL60 steel, dynamic properties

## Abstract

Urban rail wheels endure prolonged exposure to frequent starts and stops, heavy cyclic loads, and complex track conditions, which often lead to premature failure modes such as wear, fatigue cracking, and corrosion in conventional wheel materials. These limitations restrict their ability to meet the evolving demands of modern rail systems for enhanced durability and performance. To address this, the present study uses laser cladding to deposit high-entropy alloy coatings with systematically varied aluminium content onto wheel substrates. The study compares phase composition, microstructure, and mechanical properties across the different coatings. Results show that increasing Al content transforms the coating microstructure from a single face-centred cubic (FCC) phase to a dual-phase structure of FCC and body-centred cubic (BCC) phases, accompanied by notable grain refinement. Among the variants, the CrMnFeCoNi(Al)_8_ coating has the densest microstructure and the most favourable mechanical performance. It achieves a microhardness of 399.62 HV_0.5_ in the as-clad state and 450 ± 5 HV_0.5_ after heat treatment, representing an increase of approximately 12.6%. This coating also demonstrates improved corrosion resistance, with an open-circuit potential 0.07 V higher than the CL60 substrate. Multi-body dynamics simulations confirm that the clad wheels maintain excellent operational stability and safety under service conditions.

## 1. Introduction

The rapid expansion of rail transit systems has intensified wheel-rail wear and fatigue, posing a growing threat to operational safety and escalating maintenance demands [1]. Conventional repair methods are often limited by weak interfacial bonding and a tendency to crack, making it difficult to achieve an optimal balance between wear resistance, toughness, and corrosion performance [2,3,4,5]. Consequently, implementing advanced surface strengthening and repair technologies—enhancing wheel materials at a fundamental level—has emerged as an essential strategy to ensure operational safety, reduce lifecycle costs, and support the sustainable development of the railway industry [6,7,8,9,10,11].

Laser cladding is a widely adopted surface modification technique for producing protective coatings. This method forms a metallurgical bond between the coating and the substrate, yielding a dense, fine microstructure. Compared to traditional techniques, it offers advantages such as low heat input, a minimal heat-affected zone (HAZ), reduced deformation, and the ability to create a metallurgical bond between the clad layer and the substrate [12,13,14,15,16,17]. High-entropy alloys (HEAs) overcome the limitations of single-component alloys by employing multi-component designs to form simple solid solution phases [18]. Exhibiting characteristic effects such as high entropy, sluggish diffusion, severe lattice distortion, and the cocktail effect, HEAs demonstrate exceptional properties including high strength, high hardness, and outstanding oxidation and corrosion resistance [19,20,21,22,23,24,25]. The three process parameters that most significantly impact coating quality during laser cladding are laser power, scanning speed, and powder feed rate. Significant interactions among these three critical factors exist, and improper parameter settings can lead to coating defects [26,27,28]. Seo et al. [29] employed laser cladding to deposit various metallic materials onto rails, enhancing their wear resistance and comparing the effects of these materials on wheel-rail rolling contact fatigue. Ye et al. [30] employed microbeam plasma cladding to deposit CoCrFeMnNi high-entropy alloy coatings with varying aluminium contents onto substrate surfaces. They systematically investigated the effects of aluminium content on coating microstructure, wear resistance, corrosion resistance, and high-temperature oxidation resistance. Numerous studies have confirmed that aluminium content is a key factor in regulating the phase composition of high-entropy alloy coatings. Sun et al. [31] prepared CoCrFeNiAl_x_Mn_(1-x)_ high-entropy alloy coatings on the surface of AISI 1045 steel via laser cladding technology. Their research revealed that the addition of aluminium forms a dense oxide film on the material surface, enhancing its corrosion resistance. Gao et al. [32] employed laser additive manufacturing on a 45 steel substrate, simultaneously feeding powder to incorporate varying Al contents into the high-entropy alloy CoCrFeMnNi, and investigated the in situ precipitation-strengthening effect of Al. Cheng et al. [33] employed laser cladding to deposit Fe-13Cr-xAl coatings with varying Al contents on superheater surfaces, investigating the effects of Al content on coating microstructure, microhardness, and high-temperature corrosion resistance.

However, most existing studies have focused on general steel substrates or specific components, leaving a notable gap in systematic research on high-entropy alloy coatings tailored for CL60 steel—a material engineered specifically for urban rail wheels. To date, efforts to optimise aluminium content matching have yet to deliver well-rounded solutions capable of simultaneously satisfying the integrated performance demands of wheel applications, including hardness, wear, and corrosion resistance, and dynamic stability. In response, this study systematically examines the CrMnFeCoNi(Al)_x_ high-entropy alloy system. We elucidate how Al content governs the coating’s phase composition, microstructure, and mechanical properties, while employing multi-body dynamics simulations to verify the operational reliability of coated wheels.

## 2. Experimental Materials and Methods

### 2.1. Preparation of Laser Cladding Coatings

A high-entropy alloy coating composed of CrMnFeCoNi(Al)_x_ was deposited on a CL60 steel substrate by laser cladding. The cladding material was uniformly mixed from CrMnFeCoNi high-entropy alloy powder and Al powder, with Al mass fractions of 4%, 6%, and 8%, respectively. The substrate material was CL60 steel, with its chemical composition shown in Table 1.

**Figure 1:**
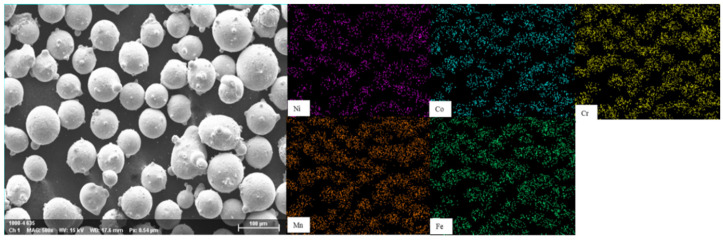
SEM morphology of CrMnFeCoNi powder.

The SEM morphology of the CrMnFeCoNi powder is shown in Figure 1. The specific chemical compositions of the high-entropy alloy powders with different Al contents are listed in Table 2.

Before laser cladding, the CL60 steel substrate was ground to a smooth finish. It was then ultrasonically cleaned with acetone for 15 min to remove oil contamination, followed by drying in an oven at 60 °C for 2 h. The cladding powder was mixed in a powder mixer for 2 h to ensure uniformity.

Based on preliminary process optimisation experiments, the following optimal process parameters were selected: laser power of 1400 W, scanning speed of 2 mm/s, powder feed rate of 20 g/min, spot diameter of 3 mm, and an overlap ratio of 30%. The cladding process was conducted under an argon protective atmosphere to prevent oxidation of the coating.

### 2.2. Microstructural Characterisation

Microstructure and composition analysis were performed using a Zeiss AxioObserver optical microscope (OM) (Carl Zeiss AG, Oberkochen, Germany) and a Thermo Scientific MIRA3 XMH scanning electron microscope (SEM) (Thermo Fisher Scientific, Waltham, MA, USA) equipped with energy-dispersive spectroscopy (EDS), enabling direct observation of grain morphology, phase distribution, and elemental uniformity within the coating. Phase composition was determined using a Cu-targeted X-ray diffractometer (XRD) (Bruker AXS, Karlsruhe, Germany) with a scanning angle range of 10° to 100°. Phase structures within the coating were identified by comparing patterns with standard PDF files (ICDD, Newtown Square, PA, USA) using MDI Jade 6.5 software (Materials Data, Inc., Livermore, CA, USA).

### 2.3. Performance Testing Methods

Using the HXD-1000TB Vickers microhardness tester (Shanghai Taiming Optical Instrument Co., Shanghai, China), measurements were taken sequentially from the surface to the substrate along the coating cross-section under a 500 g load with a 15 s dwell time. Indentation spacing was maintained at 0.1 mm to avoid work-hardening effects. Each sample was tested at 10 points in the coating, heat-affected zone, and substrate region, totalling 30 points. Prior to testing, indentation morphology was examined under an optical microscope (Olympus, Tokyo, Japan). Only indentations exhibiting regular morphology and distinct diagonal lines were statistically included using ImageJ software (v1.53, NIH, Bethesda, MD, USA). All measurements complied with requirements for indentation spacing and specimen thickness.

The GPM-40 rolling contact fatigue wear tester (Jinan Yihua Tribology Testing Technology Co., Jinan, China) was employed, utilizing U71Mn rail material (as per TB/T 2344-2012, Beijing, China) as the counter-wearing component. Both the specimen and counter-wearing roller had a radius of 30 mm, with a line contact width of 5 mm. Based on the field axle load of 14 t, the maximum wheel-rail contact stress was calculated as 1100 MPa using Hertzian contact theory. Consequently, the contact load was set at 2550 N for CL60 steel wheels and 2625 N for coated wheels. Test parameters included a slip rate of 0.75%, rotational speed of 500 rpm, and a cycle count of 2 × 10^4^ cycles. Weight measurements were taken before and after testing using a TG328A electronic balance (Shanghai Liangping Instruments Co., Shanghai, China). Mass difference was calculated to determine wear volume and wear rate. Each sample group underwent three tests, with the average values used to compare the wear performance of different wheel materials.

Using the CHI760F electrochemical workstation(CH Instruments, Inc., Shanghai, China), dynamic potential polarisation and electrochemical impedance spectroscopy (EIS) tests were conducted on the coating in a 3.5 wt.% NaCl solution under a three-electrode system. The dynamic potential polarisation scan rate was 1 mV/s, with a scan range from open-circuit potential from −0.5 V to +1.0 V. The corrosion current density (icorr) and corrosion potential (Ecorr) were determined by the Tafel extrapolation method. Linear segments of the anodic and cathodic branches, extending approximately ±100 mV from the corrosion potential, were fitted to obtain the Tafel slopes. The intersection of these extrapolated linear regions yields icorr and Ecorr. All fitting procedures followed the standard guidelines for Tafel analysis, with correlation coefficients (R^2^) exceeding 0.98 for all fitted segments, confirming the reliability of the derived parameters. The EIS test frequency range was 10^5^ Hz to 10^−2^ Hz, with a perturbation signal of 5 mV. Each sample was tested three times, with results reported as “mean ± standard deviation.” Impedance data were analysed using complex nonlinear least-squares (CNLS) fitting with an equivalent circuit programme (CHI Software, v17.04, CH Instruments, Inc., Shanghai, China).

### 2.4. Kinetic Simulation

To accurately evaluate the dynamic service performance of coated wheels, a rigid-flexible coupled dynamic model of the wheel structure was developed using the multibody dynamics simulation software Simpack (Version 2022, Dassault Systèmes, Vélizy-Villacoublay, France), in conjunction with finite element tools including HyperMesh (Version 2022.1, Altair Engineering, Inc., Troy, MI, USA) and Ansys (Version 2021 R2, Ansys, Inc., Canonsburg, PA, USA). Accounting for nonlinear wheel-rail contact and track excitation effects, the dynamic performance of coated and uncoated wheel models was compared, with particular attention to key metrics such as running smoothness, vertical and lateral stability, and wear characteristics. A systematic analysis was conducted of the dynamic response of the high-entropy alloy-clad wheel model and its influence on overall vehicle performance, thereby assessing the practical applicability of such coatings in real rail service environments.

## 3. Preparation of Laser Cladding Coatings

### 3.1. Phase Composition and Microstructural Analysis of Laser Cladding Coatings CrMnFeCoNi(Al)_x_

**Figure 2:**
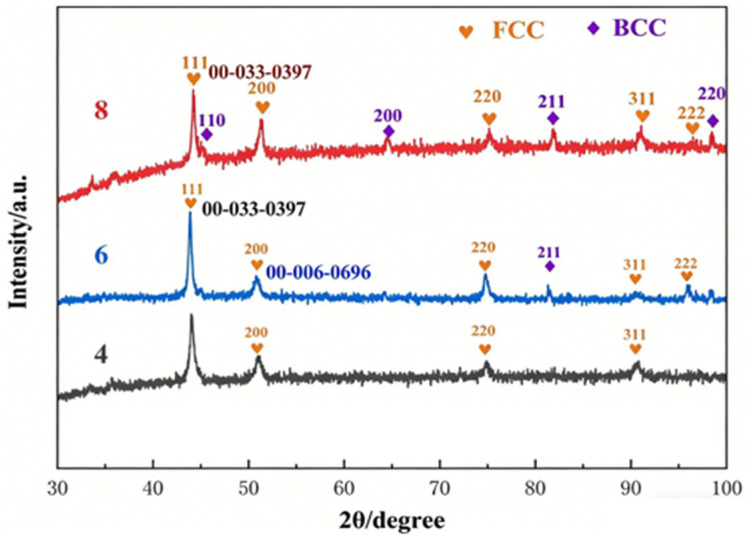
XRD detection results of CrMnFeCoNi(Al)_x_ (*x* = 4, 6, 8) high-entropy alloy cladding layer.

To verify process reproducibility, three independent batches of samples were prepared for each coating composition. Subsequent characterisation results were obtained from these three parallel batches. The X-ray diffraction (XRD) scans of the CrMnFeCoNi(Al)_x_ (x = 4, 6, 8, wt.%) high-entropy alloy coatings are shown in Figure 2. To accurately identify the phase composition of the coatings, the experimentally obtained XRD patterns were compared with JCPDS standard cards. At 4% Al content, all diffraction peaks were successfully indexed to a face-centred cubic (FCC) structure, showing good agreement with the FCC phase standard card JCPDS No. 00-033-0397. As Al content increased to 6% and 8%, new diffraction peaks appeared alongside the FCC phase peaks. These were identified as belonging to a body-centred cubic (BCC) structure, corresponding to JCPDS No. 00-006-0696, indicating the formation of a dual-phase structure (FCC + BCC) within the coating. To validate the repeatability of XRD analysis, each sample was scanned at three distinct positions, and three batches of independently prepared parallel samples were tested. All obtained XRD patterns were essentially consistent, confirming the coating’s phase composition exhibits excellent process reproducibility.

From a phase-evolution perspective, at lower Al contents, all constituent elements exhibit high mutual solubility, leading to a microstructure dominated by stable FCC solid solutions. As the Al content increases, intensified lattice distortion among solute atoms reduces the stability of the FCC phase. At the same time, the BCC structure—with an atomic packing density of 68%, lower than the 74% of FCC—provides a more accommodating environment for the larger Al atoms, thereby promoting the formation of BCC solid solutions. During laser cladding, the high-energy laser beam induces dilution, driving elements such as Fe and C from the substrate into the molten pool, thereby reducing the effective Al concentration. Additionally, due to its low melting point, Al partially evaporates under laser irradiation during powder feeding, further lowering its content in the coating. As a result, unlike high-Al high-entropy alloy powders, the coating does not develop a BCC-dominated phase structure. Nevertheless, with a controlled increase in Al content, a certain proportion of the BCC phase still forms within the coating. Given that the BCC structure generally exhibits higher strength and hardness than the FCC phase, its presence enhances the overall mechanical properties of the coating.

XRD analysis of the CrMnFeCoNi(Al)_x_ coating indicates that at an Al content of 4%, the coating exhibits a single face-centred cubic structure. Diffraction peaks appear at 43.7°, 50.9°, and 74.6°, corresponding to the (111), (200), and (220) crystal planes, respectively. When the Al content increased to 6%, a new diffraction peak appeared near 44.5°. Comparison with the JCPDS standard card assigned this peak to the (110) crystal plane of the body-centred cubic (BCC) structure. As the Al content increased to 8%, the intensity of the BCC phase diffraction peak increased significantly, indicating that the volume fraction of the BCC phase increased with increasing Al content. Gao et al. [32] observed that BCC phase formation commenced at an Al content of 5 at.% during laser additive manufacturing of CrMnFeCoNiAl_x_ alloys. In contrast, Ye et al. [30] reported that the FCC-to-BCC transformation in CoCrFeMnNiAl_x_ coatings produced via microbeam plasma cladding occurred when the Al content exceeded a 0.5 molar ratio. In this study, the BCC phase emerged at an Al content of 6 wt.%, consistent with the transformation trends reported in the aforementioned literature.

The precise 2θ positions and relative intensities of each diffraction peak are labelled in Figure 2, with the corresponding calculated lattice parameters presented in Table 3.

As shown in Table 3, with the Al content increasing from 4% to 8%, the diffraction peaks of the BCC phase gradually appear and intensify, indicating that the volume fraction of the BCC phase increases with rising Al content. The grain size of the FCC phase decreases gradually from 45.3 nm to 32.1 nm, while the BCC phase grain size also refines with increasing Al content. This grain refinement phenomenon is primarily attributed to the incorporation of Al, which increases the lattice distortion in the alloy system, thereby inhibiting grain boundary migration and grain growth. The synergistic effect of Hall-Petch strengthening from grain refinement and solid solution strengthening induced by lattice distortion significantly enhances the mechanical properties of the coating. As Al content increased from 4% to 8%, coating hardness rose from 249.64 HV0.5 ± 6.5 HV0.5 to 399.62 HV0.5 ± 8.7 HV0.5, while wear rate decreased by approximately 43.4%. These results indicate that under high-stress rolling contact conditions, the refined grain structure and lattice distortion effects effectively suppress plastic deformation and wear damage in the coating, thereby improving its tribological performance.

**Figure 3:**
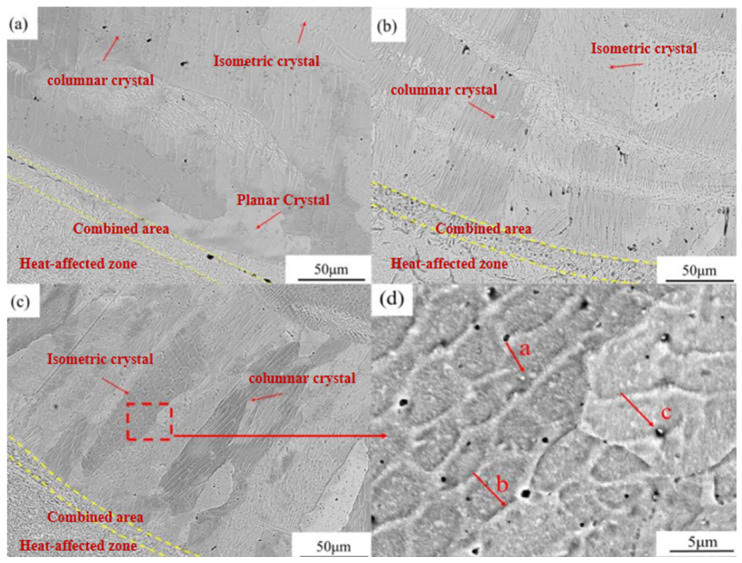
Cross-sectional SEM images at different Al contents: (**a**) CrMnFeCoNi(Al)_4_; (**b**) CrMnFeCoNi(Al)_6_; (**c**) CrMnFeCoNi(Al)_8_; (**d**) CrMnFeCoNi(Al)_8_ enlarged view.

SEM micrographs of CrMnFeCoNi(Al)_x_ (*x* = 4, 6, 8 wt.%) coatings are presented in Figure 3, where Figure 3a–c show the respective microstructures and Figure 3d provides a magnified view of the Al_8_ coating. All coatings exhibit a mix of planar, cellular, columnar, and equiaxed crystal morphologies. As illustrated in Figure 3a, the coating with 4 wt.% Al consists mainly of coarse columnar grains, with a limited amount of equiaxed crystals, and displays a relatively uniform microstructure without obvious second-phase precipitation within the grains. With increasing Al content to 6 wt.%, noticeable grain refinement occurs: the proportion of columnar crystals decreases while equiaxed crystals become more prevalent. Further increasing Al to 8 wt.% enhances this refinement trend, resulting in a microstructure dominated by equiaxed grains, with planar and columnar features substantially reduced. Additionally, as seen in the magnified view in Figure 3d, fine white nanoscale precipitates are uniformly dispersed throughout the CrMnFeCoNi(Al)_8_ coating.

Based on preliminary microstructural observations, it can be inferred that increasing Al content progressively amplifies lattice distortion in the coating. This elevated distortion increases internal strain energy, effectively suppressing grain growth and promoting a finer, more uniform microstructure dominated by equiaxed grains. Concurrently, once lattice distortion exceeds a critical threshold, BCC phases begin to precipitate within the alloy system to relieve distortion, thereby steering the system toward greater thermodynamic stability. XRD analysis confirms that the uniformly dispersed nanoscale precipitates possess a BCC structure. These precipitates act as effective pinning points, hindering dislocation motion and grain boundary migration, thereby further inhibiting grain growth and enhancing refinement. According to the Hall–Petch relationship, this grain refinement significantly improves the material’s mechanical properties [35,36,37,38].

**Figure 4:**
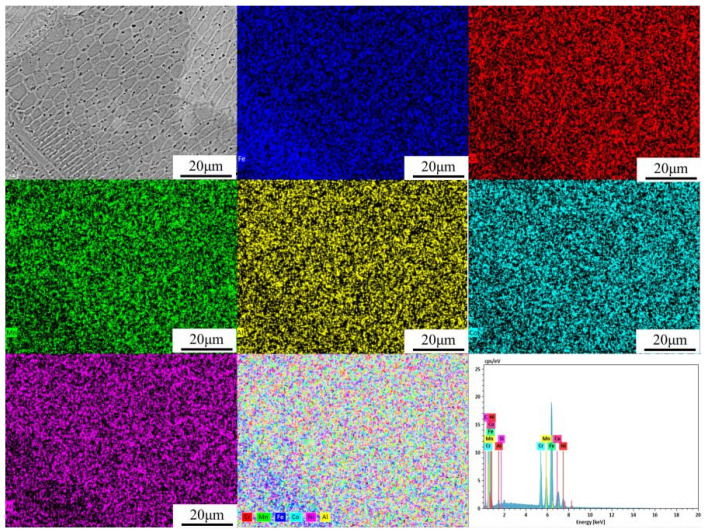
EDS surface scanning of CrMnFeCoNi(Al)_8_ cladding layer.

EDS surface scanning was conducted across the area adjacent to the bright band in Figure 3c for the three experimental cladding layers, with a 30% overlap rate, and the results are shown in Figure 4 and the quantitative data are summarised in Table 4. The analysis indicates that the Fe content in this region exceeds the theoretical value and is concentrated near the bright band, confirming that Fe is the primary component of this feature. In contrast, the Al content falls below the expected level, which can be attributed to two main factors: first, the loss of Al due to high-temperature volatilisation or oxidation during processing, leading to incomplete dissolution; and second, dilution effects from the substrate, where Fe migration into the coating under high-energy laser irradiation reduces the effective atomic percentage of Al. Consequently, despite the nominal addition of 8 wt.% Al, its actual measured content remains relatively low. This likely explains the absence of a BCC-dominated phase structure, which aligns with earlier findings [39]. To comprehensively evaluate the coating’s formation quality and reliability, quantitative characterisation was performed on the thickness uniformity, dilution ratio, porosity, and interfacial bonding quality of the CrMnFeCoNiAl_8_ coating. As shown in Figure 3d, a continuous, clean metallurgical bonded interface formed between the coating and the CL60 substrate, with no cracks or delamination defects observed, indicating excellent interfacial bonding quality. Based on measurements from five cross-sectional SEM images, the average coating thickness was approximately 450 ± 25 μm, with thickness variations at each measurement point less than 6%, indicating excellent thickness uniformity of the cladding layer. Based on the trend of Fe element content from the interface toward the coating surface, the dilution rate of the coating is estimated to be approximately 8–10%. This low dilution rate indicates a strong metallurgical bond between the coating and substrate, with minimal interference from substrate melting on the coating composition, consistent with the defect-free interface observed in Figure 3d. Using ImageJ (Version 1.53, National Institutes of Health, Bethesda, MD, USA)to evaluate the porosity of five cross-sectional photographs statistically revealed an average coating porosity of 0.4%. This exhibits a highly dense microstructure, which enhances the coating’s barrier properties and mechanical reliability. Meanwhile, the data demonstrate that principal high-entropy elements, such as Cr, Co, and Ni, exhibit consistent composition and uniform distribution across different regions, reflecting the effective alloying behaviour and compositional homogeneity achieved during the cladding process.

XRD analysis confirmed the coexistence of FCC and BCC solid-solution phases in the Al_8_ coating. To further investigate the composition of the two-phase solid solution, EDS point scans were performed at points a–c in Figure 3d. Point a is located within a grain in the centre of the coating, point b is near a grain boundary, and point c is within an agglomerate of white nanoscale precipitates. The EDS analysis results for the three points are shown in Table 5, revealing that the Al content gradually increases from 8.35% at point a to 22.92% at point c. This indicates significant segregation behaviour of Al at the micro-scale. Combined with the XRD results, the region at point a, with lower Al content, is dominated by the FCC phase, while the region at point c, with higher Al content, corresponds to the BCC phase. This aligns with the mechanism where Al promotes the formation of the BCC phase. The XRD phase identification results are further supported by EDS compositional analysis. As shown in Table 5, the region with higher Al content in the Al_8_ coating (point c) corresponds to the BCC phase, while the region with lower Al content (point a) corresponds to the FCC phase. This consistency with XRD analysis confirms the reliability of the phase identification.

Although surface EDS analyses with a depth resolution of approximately 5 μm may be affected by laser-induced thermal effects, measurements conducted under controlled conditions still provide reliable comparative trends among coatings. The observed Al loss due to volatilisation and dilution is discussed above, and the correlation between Al content and phase evolution is consistent with XRD results. Future work employing cross-sectional EDS or depth-sensitive techniques would be beneficial for further validating absolute composition and through-thickness uniformity.

### 3.2. Mechanical Properties Analysis of CrMnFeCoNi(Al)_x_ Coatings

Hardness tests were conducted on coatings with three different aluminium contents using a Vickers hardness tester (Wilson, Model 402MVD, Buehler, Lake Bluff, IL, USA, with testing software Version 1.2.3). The highest average hardness of 399.62 HV_0.5_ was achieved in the coating with 8 wt.% Al, followed by 334.54 HV_0.5_ for the 6 wt.% Al coating, and 249.64 HV_0.5_ for the 4 wt.% Al coating. This consistent increase in hardness with increasing Al content is primarily due to solid-solution strengthening. As Al atoms substitute for other principal elements in the crystal lattice, they create a highly concentrated solid solution that intensifies lattice distortion, impedes dislocation motion, and thereby enhances the overall strength and hardness of the material.

The hardness of the Al_6_ coating increases markedly compared to Al_4_ due to Al promoting the phase transformation from FCC to BCC. Experimental data show that at 6 wt.% Al, the BCC phase precipitates within the alloy. Since the BCC phase typically exhibits greater hardness and strength than the FCC phase, its formation directly enhances material hardness. As the Al content rises to 8 wt.%, the BCC phase fraction continues to increase, resulting in a peak coating hardness. This demonstrates that BCC phase formation is crucial for coating strengthening.

**Figure 5:**
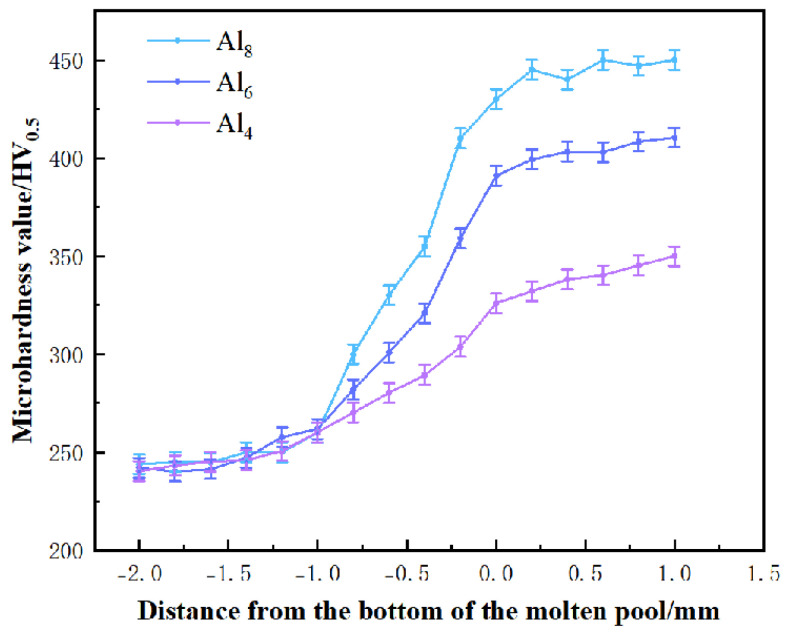
Hardness of CrMnFeCoNi(Al)_x_ overlay after heat treatment.

Test results indicate that the hardness trend of the specimens exhibits softening in the base material and a sharp increase in the HAZ. To enhance its application as a surface-strengthening coating for wheels, heat treatment was employed to mitigate the adverse effects of the HAZ, resulting in a balanced hardness and toughness across the entire specimen [40]. Figure 5 shows the hardness of the heat-treated CrMnFeCoNi(Al)_x_ coating. The heat treatment process involved holding at 850 °C for two hours, followed by oil quenching, high-temperature tempering at 550 °C for two hours, and air cooling. Following heat treatment, the hardness transition between the coating, HAZ and substrate became smoother, eliminating the abrupt hardness increase in the heat-affected zone. The coating hardness further increased, with Al_8_ exhibiting the highest hardness of 450 ± 5 HV_0.5_, approximately 1.83 times that of the substrate. The statistical distribution of hardness indicates excellent mechanical uniformity of the coating. The standard deviation of hardness values across all regions is less than 5% of the measured mean, confirming the stability of the laser cladding process and the consistency of coating properties.

Given its favourable phase composition, refined microstructure, and superior hardness, the CrMnFeCoNi(Al)_8_ coating demonstrates the most promising characteristics among the tested specimens. It was therefore selected for further evaluation of its mechanical properties as a laser-clad surface layer for wheel applications.

**Figure 6:**
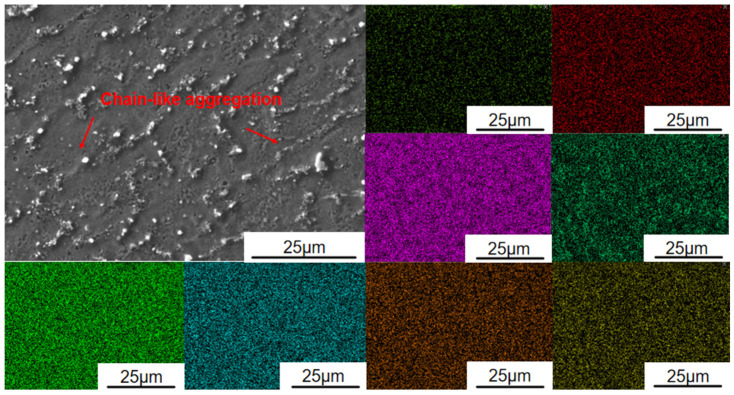
Morphology of CrMnFeCoNi(Al)_8_ under heat treatment.

Further enhancement of coating hardness is influenced by heat treatment, which promotes element diffusion within the Al_8_ coating and precipitation of BCC phase particles, as shown in Figure 6. SEM observations reveal that compared to the original grain morphology, grain boundaries become less distinct. A large number of second-phase particles precipitate along grain boundaries and aggregate in chain-like structures. These particles effectively pin the grain boundaries, restricting grain slip under external forces and thereby enhancing the material’s high-temperature stability and strength. The formation mechanism of the nanoscale precipitates in Figure 6 can be inferred from two aspects: First, Al readily oxidises at high temperatures, consistent with findings in Al-containing alloys. Second, the morphology and contrast characteristics of these fine particles under SEM correspond to those of oxides. Although TEM confirmation is unavailable, considering the morphology, distribution, and high-temperature oxidation behaviour of Al, it is inferred that these are primarily Al_2_O_3_ particles. The identification of carbides is based on the enrichment of C and Cr elements at grain boundaries in Figure 4, and the fact that this precipitation behaviour aligns with the carbide formation mechanism observed in iron-based alloys during heat treatment. Due to the lack of selected area electron diffraction (SAED) or TEM-EDS analysis, the current identification of carbides remains speculative; hence, the term “carbide-like precipitation phase” is used in the text. These oxides and carbides are predominantly hard phases. Their uniform distribution throughout the microstructure effectively acts as secondary phase strengthening, increasing the difficulty of dislocation movement and thereby enhancing the overall hardness and wear resistance of the coating.

In this study, the hardness of the Al_8_ coating after heat treatment was 450 ± 5 HV_0.5_, slightly higher than the 380–420 HV reported by Sun et al. [31]. This may be attributed to the relatively high carbon content in the CL60 steel substrate, where partial carbon diffusion into the coating during cladding formed a dispersion-strengthening phase of carbides, further enhancing hardness. The wear rate reduction of 43.4% aligns closely with the 40–50% reported by Guo et al. [41], indicating that high-entropy alloy coatings offer comparable wear resistance to traditional Co-based alloys while exhibiting superior corrosion resistance. Constrained by the complexity of the coating/substrate interface and the coating’s inherent brittleness, TEM sample preparation proved challenging. Combined with current experimental limitations, this study did not provide direct atomic-scale evidence, such as TEM or EBSD analysis. Future research should integrate high-resolution TEM and EBSD techniques to further elucidate the crystal structure and chemical composition of the precipitated phases, thereby providing a deeper understanding of the strengthening mechanism.

**Figure 7:**
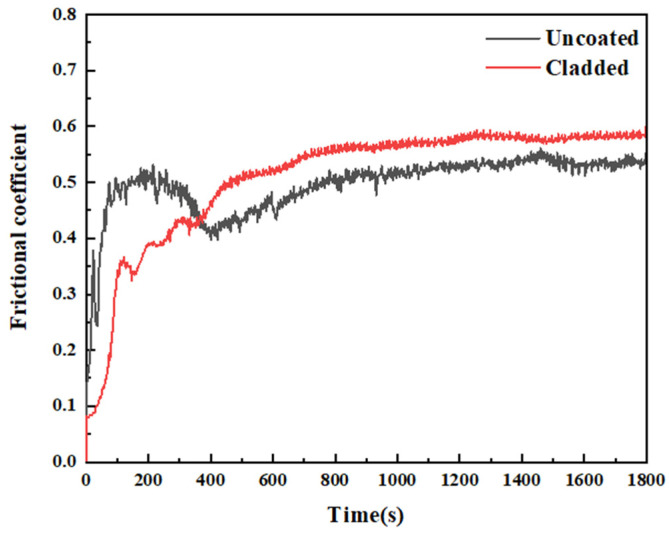
Friction coefficient of uncoated and coated specimens.

As shown in Figure 7, the rolling friction coefficients of CL60 steel and the CrMnFeCoNi(Al)_8_ coating stabilised at approximately 0.43 and 0.40, respectively. Regarding wear rate, experimental results indicate that the CL60 steel wheel exhibits a wear rate of (1.45 ± 0.12) × 10^−5^ g/m. In contrast, the laser-clad wheel reduces this to (0.82 ± 0.08) × 10^−5^ g/m—a decrease of approximately 43.4% compared to the uncoated steel wheel. This significant difference demonstrates the distinct advantage of laser cladding technology in enhancing material wear resistance. The enhanced wear resistance stems from the synergistic interaction between the coating’s microstructure and phase composition. The solid-solution strengthening effect promotes the formation of abundant BCC phases within the coating, leading to significant grain refinement. This microstructural transformation endows the coating with high microhardness. Under rolling friction conditions, the high-hardness microstructure effectively suppresses plastic deformation of the coating, substantially improving its wear resistance. Concurrently, the Al_2_O_3_ oxide film formed on the worn surface acts as a lubricating barrier, effectively preventing direct contact between the wheel and rail. This reduces both mechanical wear and adhesive wear between the wheel-rail specimens.

Based on the friction coefficient behaviour and the known wear mechanisms of similar high-entropy alloy coatings reported in the literature [42,43], the worn surface of the coated sample is expected to exhibit mild abrasive wear with occasional oxidative patches, while the uncoated CL60 steel likely shows more severe adhesive wear and plastic deformation. Future work should include systematic worn surface analysis to further elucidate the wear mechanisms.

**Figure 8:**
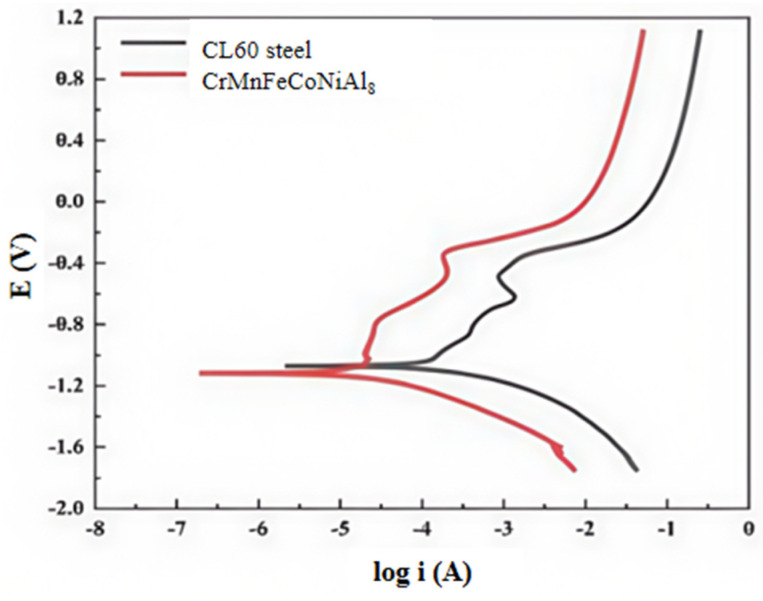
Polarisation curves of CL60 steel and high-entropy alloy cladding layer in 3.5% NaCl solution.

To comprehensively evaluate the corrosion resistance of the coating, this study employed both electrochemical impedance spectroscopy and polarisation testing to assess and compare the corrosion resistance of the CL60 steel substrate and the CrMnFeCoNi(Al)_8_ coating. Dynamic potential polarisation scans were performed on both the CL60 steel substrate and the CrMnFeCoNi(Al)_8_ high-entropy alloy coating. Electrochemical parameters-the self-corrosion potential (Ecorr), self-corrosion current density (icorr), and breakdown potential (Eb)-were determined by extrapolating the Tafel line. The Tafel extrapolation method was employed to determine the electrochemical parameters from the polarisation curves shown in Figure 8. The linear portions of both anodic and cathodic branches were clearly identifiable, enabling reliable determination of corrosion current density and corrosion potential. The resulting parameters are summarised in Table 6.

Figure 8 shows the potentiodynamic polarisation curves of the CL60 steel substrate and the CrMnFeCoNi(Al)_8_ high-entropy alloy coating in 3.5% NaCl solution, with the fitted parameters listed in Table 6. Specifically, the Al_8_-coated sample exhibits a clear passive region in the NaCl solution. Compared with the CL60 substrate, the CrMnFeCoNi(Al)_8_ coating significantly suppresses corrosion, as evidenced by a much lower corrosion current density. Furthermore, the positive shift in breakdown potential suggests that the passive film on the coating surface is more stable and can withstand harsher electrochemical conditions.

**Figure 9:**
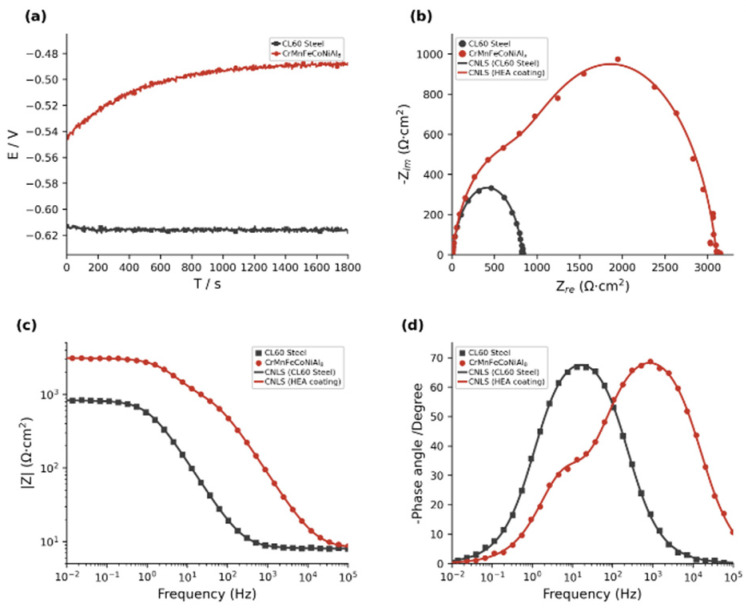
Electrochemical impedance spectroscopy analysis of CL60 steel substrate and CrMnFeCoNi(Al)_8_ coating in 3.5 wt.% NaCl solution: (**a**) Open—circuit potential versus time; (**b**) Nyquist plots; (**c**) Bode magnitude plots; (**d**) Bode phase angle plots.

The open-circuit potential and EIS results for the coating and substrate in 3.5 wt.% NaCl solution is shown in Figure 9. As seen in Figure 9a, the coating exhibits a positive shift in open-circuit potential relative to the substrate, reflecting its reduced corrosion tendency and higher thermodynamic stability. As shown in Figure 9b, the Nyquist plot of the coated sample exhibits two flattened semicircular arcs. The symbols represent experimental data, while the solid lines correspond to CNLS-fitted curves obtained using the equivalent circuit model. The excellent agreement between the experimental data and fitted lines, with χ^2^ values in Table 7 on the order of 10^−3^–10^−4^, confirms the validity of the chosen equivalent circuit. The high-frequency arc reflects the dielectric and barrier properties of the coating, characterised by R*_f_* and CPE*_f_*; the low-frequency arc corresponds to the charge-transfer process at the substrate/electrolyte interface, characterised by R*_ct_* and CPE*_dl_*. The flattened semicircular arcs indicate surface irregularities or roughness on the electrode. Using a constant-phase-angle element provides a more accurate description of this non-ideal capacitive behaviour. Bode magnitude and phase angle plots for both specimens are given in Figure 9c,d, where symbols denote experimental data and solid lines represent CNLS fits. In electrochemical impedance spectroscopy, the shielding performance of coatings against corrosive solutions is typically evaluated using the impedance at 0.01 Hz as a key metric. The impedance modulus increased from 82.76 Ω·cm^2^ for the substrate to 103.28 Ω·cm^2^, accompanied by a higher peak phase angle and a markedly broader peak width. These features indicate that a denser, more charge-retentive passive film has formed on the coating surface, which more effectively hinders the ingress of corrosive species and thus delivers significantly improved overall corrosion resistance compared with the CL60 steel substrate.

Electrochemical impedance spectroscopy results further elucidate the corrosion protection mechanism of the CrMnFeCoNiAl_8_ coating. Although the coating exhibits a higher low-frequency impedance modulus than the substrate at 0.01 Hz, the more critical parameter for assessing corrosion resistance is the R*_c__t_* derived from equivalent circuit fitting, which directly reflects the ease of charge transfer at the electrode/electrolyte interface. As shown in Table 7, the coating exhibits an Rct value of 3079 Ω·cm^2^, approximately four times that of the substrate. This significant improvement indicates the coating effectively suppresses interfacial charge transfer, substantially reducing the corrosion rate. Estimations based on the corrosion current density icorr measured via dynamic polarisation reveal that the coating reduces the corrosion rate by approximately 86% compared to the CL60 substrate. The improvement in Rct is primarily attributed to the formation of a dense passivation film dominated by Al_2_O_3_ on the coating surface. This film effectively blocks the penetration of corrosive ions such as Cl^−^. Additionally, the BCC phase structure and refined grain structure within the Al_8_ coating contribute to the formation of a more uniform and defect-free passivation film, further enhancing its protective properties. Electrochemical impedance spectroscopy fitting results indicate that all fitted chi-squared values (χ^2^) fall within the range of 10^−3^ to 10^−4^, demonstrating high fitting accuracy between the selected equivalent circuit and experimental data, and confirming the reliability of the fitting results.

### 3.3. Kinetic Analysis

**Figure 10:**
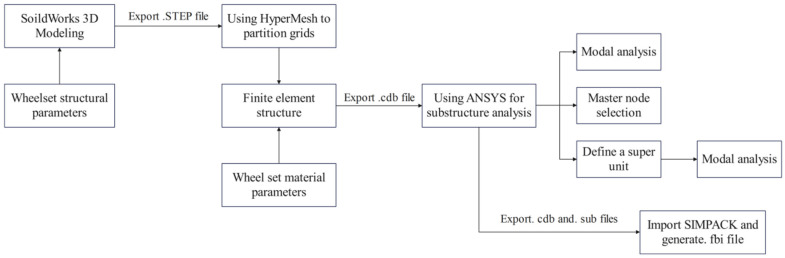
Rigid body wheelset model.

Following the simulation test conditions outlined in GB5599-2019 [44], a rigid-flexible coupled dynamic model was developed, focusing on a flexible wheelset and a flexible bogie frame. The preparatory steps for the rigid-flexible coupling analysis were carried out in SolidWorks, HyperMesh, and Ansys in sequence to generate the meshed geometry and substructure files. These files were then imported into the Utilities module of Simpack to create the flexible body components. The overall workflow is illustrated in Figure 10.

**Figure 11:**
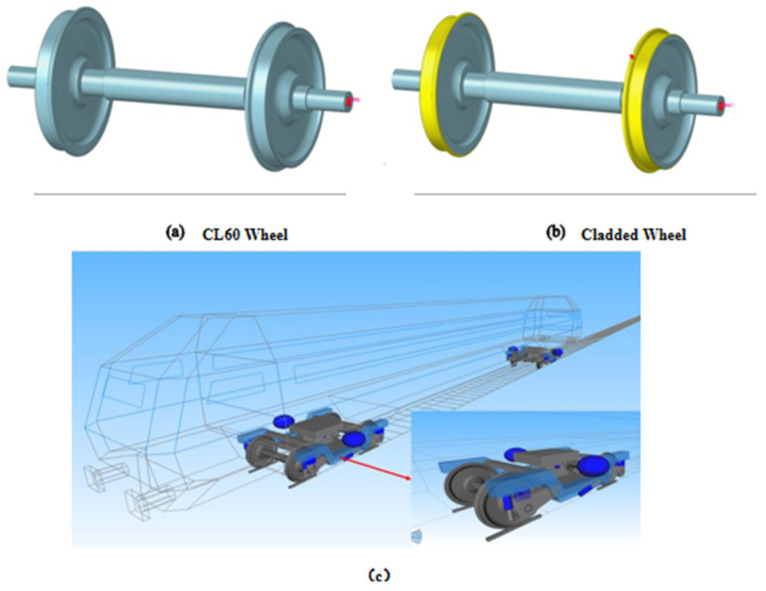
Schematic and Model of Wheel Surface Material Evolution: (**a**) CL60 steel wheel; (**b**) cladding-coated wheel model; (**c**) Rigid-flexible coupling dynamics model.

A three-dimensional solid wheelset model was initially developed in SolidWorks and then imported into HyperMesh for meshing and material property definition. In the unclad configuration, all elements had CL60 steel properties, as shown in Figure 11a. In contrast, for the clad wheelset, the core remained CL60 steel, while the external coating region is assigned corresponding material properties, as illustrated by the yellow sections in Figure 11b. The required material properties—elastic modulus, Poisson’s ratio, and density—were measured experimentally, with the specific values for both CL60 steel and the coating listed in Table 8. Finally, the resulting coupled dynamic model, incorporating both rigid and flexible components, is shown in Figure 11c.

Based on Simpack simulation analysis, this paper evaluates the impact of laser-cladding coatings on train running smoothness, using the left wheel of the previous bogie as an example. It compares the lateral and vertical smoothness metrics of CL60 steel wheels and coated wheels at different speeds. Therefore, this study directly calculates the Sperling smoothness evaluation metric in Simpack’s post-processing software, using the formula given in (1). The software employs a continuous single weighting function and corresponding integration algorithm to reliably determine the vehicle body’s lateral and vertical ride comfort.

(1) $$W_{i}=3.57\sqrt[{10}]{\frac{A_{i}^{3}}{f_{i}}F(f_{i})}$$

where i = 1, 2, 3, …, n;

$A_{i}$ is the vibration acceleration, unit m/s^2^;

$f_{i}$ is the vibration frequency, unit Hz; 0.5 Hz ≤ f ≤ 40 Hz;

F($f_{i}$) is the frequency correction factor;

$W_{i}$ is the smoothness index component at frequency $f_{i}$.

The smoothness rating table for passenger coaches and EMUs is shown in Table 9.

Simulation results indicate that the lateral and vertical ride comfort indices for both CL60 steel wheels and coated wheels at four different speeds are summarised in Table 10. The lateral comfort indices for both wheel types remain well below 2.50. Even the maximum vertical comfort index under peak vibration acceleration reaches only 2.47, which still falls within the 2.50 threshold, confirming excellent ride smoothness. Differences in lateral comfort between coated and uncoated wheels are less than 0.01, while vertical comfort differs by less than 0.03. These findings demonstrate that the coating does not compromise ride quality and contributes to a modest improvement in vertical stability.

The derailment coefficient, defined as the instantaneous ratio of lateral to vertical force on one side of a wheelset, serves as a fundamental criterion for assessing derailment risk. It evaluates whether a wheel flange will climb over the rail head under lateral loading—a critical failure mode with serious operational consequences. Given the complexity of real-world conditions and the severe hazards associated with derailment, this coefficient is a key indicator of running safety. It is calculated as follows:

(2) $$\frac{Q}{P}=\frac{\tan\alpha -\mu }{1+\mu \tan\alpha }$$

where *Q* is the lateral force acting on the flange;

*P* is the vertical force acting on the wheel;

*α* is the flange angle;

*μ* is the coefficient of friction between the flange and the rail.

This study employs a radius of R = 900 m, with an evaluation criterion of Q/P ≤ 0.8. After post-processing calculations using Simpack software, the maximum derailment coefficients for both models were compiled, with the data presented in Table 11.

The derailment coefficients for both wheel types remained below 0.4, well under the safety limit of 0.8. In both models, the coefficient decreased with increasing speed, with the coated wheels exhibiting a slightly higher value than the CL60 steel wheels. Nonetheless, all values stayed far below the safety threshold and showed stable variation throughout the test, confirming the operational safety and reliability of the coated wheels.

The wheel load reduction rate is a critical indicator that reflects the dynamic changes in wheel–rail contact conditions. It quantifies the risk of reduced vertical axle load or even momentary loss of contact due to factors such as track excitation, vehicle vibration, or dynamic mismatch at the wheel–rail interface. Elevated values may lead to wheel–rail slip, bounce, or derailment, thereby compromising both operational safety and ride comfort. In China, the current safety standard for wheel load reduction rate stipulates that when the test speed v ≤ 160 km/h, the average wheel load shall satisfy the expression given in Equation (3). The formula for calculating the average wheel load is provided in Equation (4).

(3) $$\frac{\Delta P}{P}\leq 0.65$$

(4) $$\bar{P}=\frac{1\times P_{1}+2\times P_{2}+4\times P_{3}}{8}\times 9.81$$

where $\bar{P}$ is the average wheel load, in kN;

Δ*P* denotes the wheel load reduction, measured in kN;

*P*_1_ represents the car body, *P*_2_ the frame, and *P*_3_ the wheelset, all in kN.

The maximum wheel load reduction rates for both models at various speeds are summarised in Table 12.

The load reduction rates for both wheel types remained below the specified limit of 0.65. The coated wheels recorded a marginally lower rate than the CL60 steel wheels, reflecting their improved vertical adhesion and contact stability. The reduction rate peaked within the speed range of 90–105 km/h and subsequently declined at higher speeds. Overall, the coating provided the most pronounced reduction effect at moderate speeds, contributing to enhanced operational smoothness and safety. These results suggest favourable engineering applicability under medium- to high-speed service conditions.

Axle lateral force, defined as the lateral force exerted on a wheel or axle perpendicular to the direction of travel, is a key parameter influencing vehicle stability and wheel–rail interaction. Excessive lateral force can cause snake instability, increasing the risk of rail climbing and derailment, thus necessitating its limitation. The evaluation of axle lateral force follows Equation (5), which relates the measured lateral force to the static axle load.

(5) $$H\leq 15+P_{0}$$

where *P*_0_ represents the static axle load of the wheel, measured in kN.

According to Equations (4) and (5), the allowable limit for axle lateral force is H ≤ 30.0549204 kN for CL60 steel wheels and H ≤ 30.05322 kN for coated wheels. The maximum lateral forces obtained from both wheel models across different speeds are summarised in Table 12. As shown, the values for both CL60 steel and coated wheels remain well below their respective limits, confirming that all results fall within the safe operating range. For a clearer comparison, the trend of the maximum lateral force with speed is plotted in Table 13.

**Figure 12:**
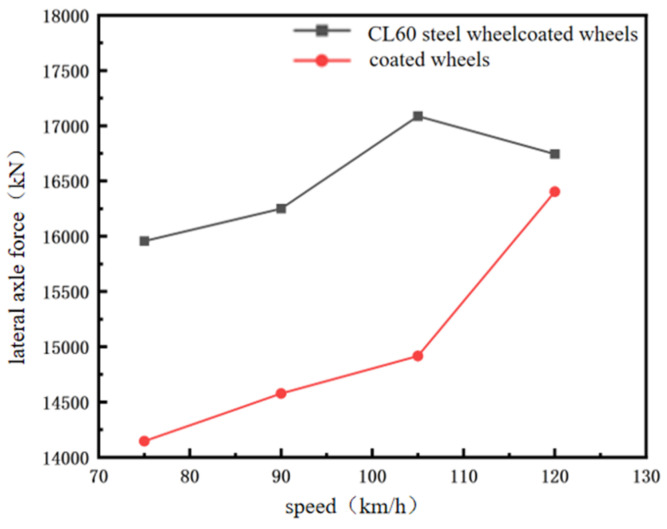
Comparison of Axle Lateral Forces.

As shown in Figure 12, the lateral force on the axle of coated wheels is lower than that of CL60 steel wheels. At low speeds, the coating dampens the dynamic response to track irregularities, thereby reducing wheel–rail interaction forces and improving lateral stability and safety. While this mitigating effect diminishes somewhat at higher speeds, it remains stable and beneficial. Integrating these observations with earlier analyses of the derailment coefficient and wheel load reduction rate, the lower lateral force of coated wheels contributes to enhanced vertical stability and rail-adhesion performance. Concurrently, the generally reduced wheel load loss leads to smoother wheel–rail contact and lowers the risk of momentary loss of contact. Overall, the reduction in axle lateral force alleviates lateral wheel–rail impacts, mitigates flange wear, extends wheelset service life, and supports suitability for urban rail operating profiles characterised by frequent stops/starts and tight curve radii.

To assess the robustness of the dynamic simulation results, a sensitivity analysis was conducted on key input parameters. The following parameters were varied by ±10% from their baseline values to observe changes in the derailment coefficient, wheel load reduction rate, and lateral wheel-axle force: coating density 7.50 ± 0.75 g/cm^3^, coating elastic modulus 200 ± 20 GPa, wheel-rail the friction coefficient 0.3 ± 0.03, and track irregularity amplitude scaled by ±10%. Analysis revealed that model outputs were most sensitive to track irregularities and the friction coefficient. When these parameters varied by ±10%, the derailment coefficient fluctuated by 8–12%. In contrast, model outputs showed low sensitivity to moderate fluctuations in coating material parameters. Changes in coating density or elastic modulus by ±10% resulted in less than 3% variation in all dynamic metrics. Under all parameter variations, coated wheels consistently outperformed uncoated wheels in wear resistance and lateral force reduction. This confirms the robustness of the study’s conclusion that coating enhances wheel performance, even in the presence of minor measurement errors in material parameters. Detailed sensitivity analysis results are presented in Table 14.

The sensitivity analysis above not only validates the robustness of the conclusions but also indirectly demonstrates that the model’s response to parameter variations aligns with physical expectations. The model exhibits high sensitivity to track irregularities and friction coefficients, consistent with the characteristics of actual wheel-rail systems. Conversely, its lower sensitivity to coating material parameters indicates that the conclusions regarding coating-enhanced wheel performance presented in this paper are reliable.

### 3.4. Wear Volume Analysis

The occurrence and progression of wear not only affect the smoothness and comfort of train operation but also directly impact equipment safety and operational maintenance costs. In wheel-rail system wear prediction, the Archard wear model is widely adopted due to its clear theoretical basis and well-defined parameters [45,46]. This model, grounded in adhesive wear theory, posits that wear volume is proportional to contact load and sliding distance while being inversely proportional to material hardness, as expressed in Equation (6).

(6) $$V=\frac{K\times F\times S}{H}$$

**Figure 13:**
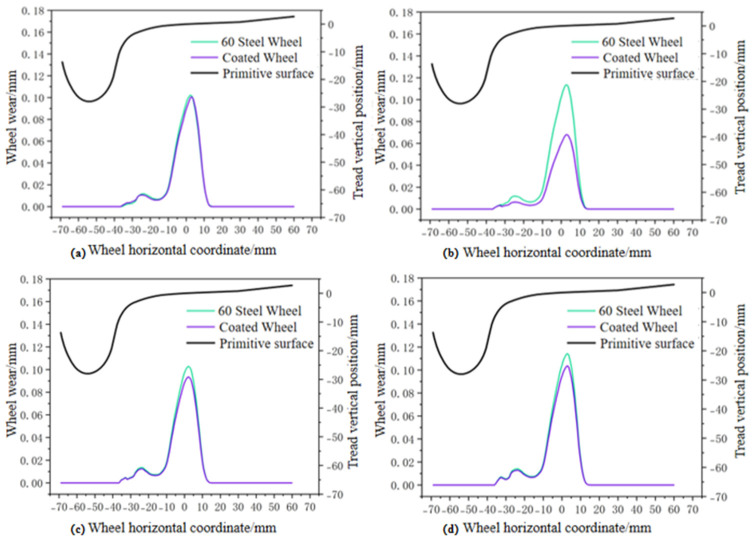
Wheel tread wear at different speeds: (**a**) 75 km/h; (**b**) 90 km/h; (**c**) 105 km/h; (**d**) 120 km/h.

*V*: Wear volume (mm^3^); *K*: Dimensionless wear coefficient, reflecting material wear tendency; *F*: Normal contact force (N); *S*: Slip distance (m); *H*: Material hardness (MPa). When simulating wear comparisons over the same distance at different speeds, the track mileage was set to 100,000 km, with speeds set at 75 km/h, 90 km/h, 105 km/h, and 120 km/h, respectively. The final wear comparison is shown in Figure 13. Figure 13 demonstrates that at all four speeds, the wear volume of the clad wheels is lower than that of the original wheel model. This indicates that the coating enhances the surface hardness of the wheels, thereby improving their wear resistance.

**Figure 14:**
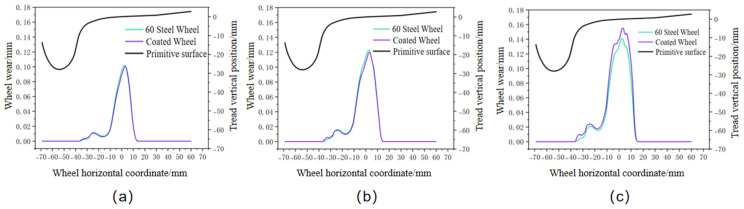
Wheel wear at different mileages: (**a**) 100,000 km; (**b**) 120,000 km; (**c**) 140,000 km.

When simulating wear comparisons at the same speed but different mileage intervals, a speed of 75 km/h was selected. Both the CL60 steel wheels and the clad wheels were configured with varying mileage settings. Since the overhaul cycle for urban rail vehicles is typically set at 140,000 km, this study set track lengths of 100,000 km, 120,000 km, and 140,000 km. The tread was replaced every 20,000 km of operation. The final wear comparison is shown in Figure 14. Wear data is presented in Table 15.

As shown in the table data, both CL60 steel wheels and coated wheels exhibit increasing wear rates with extended operating mileage. Under identical operating conditions, all three sets of data indicate that coated wheels demonstrate lower wear rates than CL60 steel wheels. The maximum wear reduction achieved by coated wheels reaches 9.09%, indicating superior wear resistance and stability over extended service periods.

Based on the combined results of two wear simulations, the coating’s wear volume was consistently lower than that of the CL60 steel wheel. This is attributed to the coating’s high hardness, which effectively resists wear under rolling contact. Additionally, its surface features—including equiaxed crystals and dispersed hard phases—further inhibit wear. Simultaneously, the coating’s lower elastic modulus and density mitigate transient impact loads during wheel-rail contact. This enhances the synergistic deformation capability between the coating and the wheel substrate, achieving a balance between hardness and toughness. Consequently, the coating demonstrates superior wear suppression performance during long-term operation.

## 4. Conclusions

This work focuses on the surface modification of urban rail vehicle wheels. Utilising laser cladding technology, high-entropy alloy coatings of CrMnFeCoNi(Al)_x_ with varying aluminium contents were fabricated on a CL60 steel substrate. The effects of aluminium content on coating microstructure, mechanical properties, and wheel dynamic behaviour were systematically investigated. The principal conclusions are summarised as follows:

(1) The addition of Al plays a decisive role in modulating the coating’s microstructure and properties. As the Al content increases from 4% to 8%, progressive grain refinement is observed. Beyond 6% Al, the microstructure transitions from a single FCC phase to a dual-phase FCC/BCC structure, which significantly enhances hardness, strength, and resistance to plastic deformation under high loads. This improvement is attributed to the synergistic effects of solution strengthening, grain refinement, and lattice distortion.
(2) In terms of mechanical performance, the coating with 8% Al content delivered the most superior results. It achieved a microhardness of 399.62 HV_0.5_ in the as-clad state, which increased to 450 ± 5 HV_0.5_ after heat treatment—approximately 1.83 times that of the substrate. Concurrently, this coating exhibited a wear rate approximately 43.4% lower than the CL60 steel substrate in rolling contact fatigue tests, confirming its excellent wear resistance. Electrochemical testing further revealed a significant enhancement in corrosion resistance within a 3.5% NaCl solution, characterised by a notably reduced corrosion current density, improved stability of the passive film, and an overall protective performance surpassing that of the base material.
(3) Dynamic simulation analysis confirms that the coated wheels improve vehicle stability. Although the derailment coefficient remains marginally higher than that of CL60 steel wheels, it stays well below the safety limit of 0.8. The maximum wheel load reduction rate and wheel–axle lateral force were reduced by 7.25% and 12.7%, respectively. Wear performance also showed notable improvement: at the same mileage and speed, wear decreased compared to the original CL60 steel wheels, and at the same speed across varying mileages, the wear rate was significantly lower, achieving a maximum wear reduction of 9.09%.
(4) From an engineering application perspective, the CrMnFeCoNiAl_8_ coating significantly enhances wheel hardness, wear resistance, and corrosion resistance, reducing wear rate by 43.4% and corrosion rate by approximately 86%. This holds promise for extending wheel reconditioning intervals and lowering full-life maintenance costs. However, this study represents only a preliminary laboratory evaluation. Further validation is required under complex service conditions involving variable loads, humidity, and deicing salts. Attention should also be paid to the long-term stability of the coating-substrate interface and degradation behaviour under rolling contact fatigue. Although the kinetic model has been indirectly validated through standard compliance, experimental parameter input, trend consistency, and sensitivity analysis, direct validation via full-scale bench or field measurements is still lacking. Future work should further calibrate the model’s accuracy using actual measurement data.

This paper presents an integrated material-process solution for surface strengthening of urban rail wheels, offering both superior mechanical properties and dynamic stability. Future research may further optimise aluminium content gradients and heat-treatment processes to expand the application of high-entropy alloy coatings under varying load levels and complex track conditions. Concurrently, exploring the combined application of laser cladding with other surface modification techniques will provide more comprehensive technical support for achieving extended service life and low-cost maintenance of critical rail transit components.

## Figures and Tables

**Table 1:** Chemical Composition of Base CL60 Steel (wt.%) [34].

Materials	C	Si	Mn	P	S	Fe
CL60	0.55–0.65	0.17–0.37	0.50–0.80	0.035	0.040	Bal.

**Table 2:** Chemical composition of high-entropy alloy powder (wt.%).

Element	Cr	Mn	Fe	Co	Ni	Al
CrMnFeCoNi	18.54	19.58	19.92	21.03	20.93	0
CrMnFeCoNi(Al)_4_	17.81	18.81	19.12	20.18	20.10	4
CrMnFeCoNi(Al)_6_	17.44	18.42	18.72	19.76	19.67	6
CrMnFeCoNi(Al)_8_	17.07	18.02	18.33	19.34	19.26	8

**Table 3:** Crystallite size and lattice parameters of CrMnFeCoNi(Al)_x_ coatings.

Al Content (wt.%)	Phase	Crystallite Size (nm)	Lattice Parameter (Å)
4	FCC	45.3 ± 3.2	3.592 ± 0.005
6	FCC + BCC	38.7 ± 2.8 (FCC)/28.4 ± 2.1 (BCC)	3.603 ± 0.006 (FCC)/2.876 ± 0.004 (BCC)
8	FCC + BCC	32.1 ± 2.5 (FCC)/23.6 ± 1.9 (BCC)	3.611 ± 0.005 (FCC)/2.882 ± 0.004 (BCC)

**Table 4:** EDS results of CrMnFeCoNi(Al)_8_ cladding layer (atomic percentage, %).

Atomic Percentage (%)	Cr	Mn	Fe	Co	Ni	Al
Theoretical Value	18.24	17.26	16.98	16.09	16.16	15.28
Actual Value	14.73	15.22	30.25	14.25	15.44	10.11

**Table 5:** EDS results of spot scanning of CrMnFeCoNi(Al)_8_ cladding layer (at.%).

Dot	Cr	Mn	Fe	Co	Ni	Al
a	19.03	17.2	20.90	17.31	17.21	8.35
b	14.76	17.68	20.89	17.74	17.32	11.39
c	10.78	13.90	18.9	14.8	18.70	22.92

**Table 6:** Polarisation curve fitting results.

Sample	Ecorr/(V)	Icorr/(μA·cm^−2^)	Eb/(V)
60 Steel Matrix	−1.064 ± 0.012	100.9 ± 8.5	−0.388 ± 0.015
CrMnFeCoNi(Al)_8_ Coated Specimen	−1.115 ± 0.009	14.4 ± 1.2	−0.326 ± 0.008

**Table 7:** Fitting results of electrochemical impedance spectroscopy.

Sample	Rs (Ω·cm^2^)	Cf (μF·cm^2^)	nf	Rf (Ω·cm^2^)	Cd (μF·cm^2^)	nd	Rct (Ω·cm^2^)	χ^2^
60 Steel Matrix	6.33 ± 0.21	——	——	——	860.8 ± 42.5	0.827 ± 0.015	760.5 ± 42.3	1.2 × 10^−3^
CrMnFeCoNi(Al)_8_	14.42 ± 0.35	265.2 ± 18.6	0.722 ± 0.012	56.91 ± 4.8	115.1 ± 9.3	0.842 ± 0.008	3079 ± 156	9.8 × 10^−4^

**Table 8:** Material properties of CL60 steel and cladding layer.

Material	Density (g/cm^3^)	Young’s Modulus (GPa)	Poisson’s Ratio
Coating	7.50	200	0.28

**Table 9:** Table of stability index grades for passenger cars and multiple units.

Stability Level	Stability Index W	Evaluation
Level 1	≤2.5	Excellent
Level 2	2.5~2.75	Good
Level 3	2.75~3	Pass

**Table 10:** Comparison of Sperling indicators of car bodies.

Speed (km/h)	Lateral Stability Index		Vertical Stability Index	
	60 Steel Wheel	Coated Wheel	60 Steel Wheel	Coated Wheel
75	1.22	1.22	2.22	2.19
90	1.31	1.30	2.47	2.47
105	1.37	1.36	1.96	1.95
120	1.43	1.43	1.95	1.94

**Table 11:** Comparison of derailment coefficients of car bodies.

Speed (km/h)	Derailment Coefficient Q/P	
	60 Steel Wheel	Coated Wheel
75	0.3555	0.3941
90	0.3602	0.3992
105	0.3518	0.3886
120	0.3308	0.3805

**Table 12:** Comparison of vehicle wheel load reduction rates.

Speed (km/h)	Wheel Load Reduction Rate	
	60 Steel Wheel	Coated Wheel
75	0.2612	0.2585
90	0.3844	0.3696
105	0.3877	0.3596
120	0.3578	0.3458

**Table 13:** Comparison of lateral forces on wheel axles.

Speed (km/h)	Axle Lateral Force (kN)	
	60 Steel Wheel	Coated Wheel
75	15.96 ± 0.42	14.15 ± 0.38
90	16.25 ± 0.45	14.58 ± 0.41
105	17.09 ± 0.51	14.92 ± 0.44
120	16.74 ± 0.48	16.40 ± 0.46

**Table 14:** Sensitivity analysis results of key parameters on dynamic indicators.

Parameter Variation (±10%)	Derailment Coefficient Variation (%)	Wheel Load Reduction Rate Variation (%)	Axle Lateral Force Variation (%)
Coating Density	1.8	2.1	1.5
Coating Elastic Modulus	2.3	2.5	2.0
Wheel-Rail Friction Coefficient	8.5	9.2	7.8
Track Irregularity	10.2	11.5	9.6

**Table 15:** Wheel wear at different mileage.

Mileage/km	100,000 km		120,000 km		140,000 km	
	60 Steel Wheel	Coated Wheel	60 Steel Wheel	Coated Wheel	60 Steel Wheel	Coated Wheel
wear volume /mm	0.1020	0.1003	0.1228	0.1198	0.1552	0.1411

## Data Availability

The original contributions presented in this study are included in the article. For further inquiries, please contact the corresponding author.
